# Physical constraints in polymer modeling of chromatin associations with the nuclear periphery at kilobase scale

**DOI:** 10.1080/19491034.2020.1868105

**Published:** 2021-01-12

**Authors:** Annaël Brunet, Nicolas Destainville, Philippe Collas

**Affiliations:** a Department of Molecular Medicine, Institute of Basic Medical Sciences, Faculty of Medicine, University of Oslo, Oslo, Norway; b Laboratoire De Physique Théorique, IRSAMC, Université De Toulouse (UPS), CNRS, Toulouse, France; c Department of Immunology and Transfusion Medicine, Oslo University Hospital, Oslo, Norway

**Keywords:** Polymer modeling, kinetic Monte Carlo, nuclear lamina, chromatin, lamin-chromatin interaction

## Abstract

Interactions of chromatin with the nuclear lamina imposes a radial genome distribution important for nuclear functions. How physical properties of chromatin affect these interactions is unclear. We used polymer simulations to model how physical parameters of chromatin affect its interaction with the lamina. Impact of polymer stiffness is greater than stretching on its configurations at the lamina; these are manifested as trains describing extended interactions, and loops describing desorbed regions . Conferring an attraction potential leads to persistent interaction and adsorption-desorption regimes manifested by fluctuations between trains and loops. These are modulated by polymer stiffness and stretching, with a dominant impact of stiffness on resulting structural configurations. We infer that flexible euchromatin is more prone to stochastic interactions with lamins than rigid heterochromatin characterizing constitutive LADs. Our models provide insights on the physical properties of chromatin as a polymer which affect the dynamics and patterns of interactions with the nuclear lamina.

## Introduction

In the mammalian nucleus, interactions of chromatin with the nuclear lamina at the nuclear periphery (NP) provide one mechanism of spatial and temporal control of DNA replication and gene expression [1]. The nuclear lamina is a meshwork of A-type lamins (lamins A and C) and B-type lamins (lamins B1 and B2) which together provide structural rigidity, mechano-resistance and regulatory functions to the nucleus [2]. Chromatin–lamina interactions are mediated by lamina-associated domains (LADs), regions of typically tens of kilobases (kb) to several megabases, enriched in silent heterochromatin [3]. Chromatin is also anchored to the nuclear envelope via interactions with integral proteins of the inner nuclear membrane [4]. This apparent redundancy in the anchoring of chromatin to the nuclear periphery, together with the tissue-specificity of several inner nuclear membrane proteins [4], the cell type-specific positioning of chromosomes at the nuclear periphery [5] and diseases linked to mutations in nuclear envelope proteins [6] underscores the importance of maintaining a proper radial organization of the genome [1,3,7].

LADs form in both nonrandom and random manners leading to constitutive LADs (cLADs) conserved across cell types, and facultative LADs [8], also called variable LADs (vLADs) [9], that show some cell type-specificity [8] in response to differentiation [10–12]. Whereas cLADs appear to be structurally important for nuclear architecture, a proportion of vLADs, such as those associated with lamin A/C, may play a role in regulating gene expression in euchromatin contexts [13–15]. Nevertheless, some vLADs may arise from spurious lamin-chromatin contacts, which both imaging and sequencing data at the single-cell level [16,17] and computational modeling [18,19] show can vary greatly between cells in a population.

Recent findings picture LADs as structurally and functionally heterogeneous, with sub-domains escaping the overall repressive LAD environment. A number of promoters within LADs are active while others are constitutively or facultatively repressed [20]. Conceptually, LAD sub-domains may emerge from distinct chromatin states [20] and from chromatin micro-loops not bound per se to nuclear lamins [21]. Supporting the latter, variability in the level of local lamin enrichment levels within LADs, including local lamin depletions, is observed not only in cell-ensemble LAD data [8,18,22,23] but also in single-cell analyses of lamin B1–chromatin interactions [17]. These observations suggest dynamic associations of chromatin with nuclear lamins. However, the physical processes driving lamin–chromatin interactions remain largely unexplored.

One strategy to address this issue is by computational modeling of chromatin-nuclear lamin interactions. Modeling has been stimulated in attempts to explain large-scale detachments of chromatin from the nuclear lamina have been observed. For instance, reduction of lamin B1 levels during senescence is associated with a loss of heterochromatin at the nuclear periphery [24–26] including a loss of lamin B1–chromatin interactions [25,27,28]. In ‘inverted’ nuclei where heterochromatin aggregates in the nucleus center while euchromatin localizes toward the periphery, heterochromatic LADs are also lost due to the reduction or absence of lamin A/C and/or lamin B receptor (LBR), an integral protein of the nuclear envelope [29]. Polymer simulations at chromosome (megabase) scale predict that a chromatin polymer interacting with a surface representing the nuclear lamina can drive compaction of attached topological domains [30]. Block co-polymer models further show that this process is favored by homotypic interactions between heterochromatic domains [31]; interestingly, models of inverted nuclei recapitulate chromatin inversions and the loss of peripheral heterochromatin [31]. Polymer models can also provide a physical explanation for phase transitions promoting contact or dissociation of chromatin with/from the nuclear lamina [32]. They also infer that regions of euchromatin can be dragged alongside heterochromatin and be co-adsorbed onto the interacting surface [32]; this could provide one explanation for the heterogeneity in the sequence and chromatin composition of LADs [20]. Moreover, by switching off an attraction strength between a chromatin polymer and the interacting surface, polymer models predict a decondensation of chromatin after release from the lamina manifested by lost or weakened polymer interactions [30,31,33]. This however seems to depend on the scale of the observation (sub-megabase vs. tens of megabases) and parameters of the models [33].

Simulations of polymer interactions with a surface provide insights into the properties of chromatin as a function of its interaction with the nuclear lamina. However, the genomic scaling of these models provides no indication on the physical configuration of the chromatin polymer at the interaction surface at more local levels, and hence on the heterogeneity of chromatin configurations at a sub-LAD level. Here, we used a modeling approach to identify, a sub-LAD scale, physical parameters of a chromatin chain influencing recruitment and stable association of domains with the nuclear lamina.

## Materials and methods

### A coarse-grained model of chromatin

**Figure 1.**
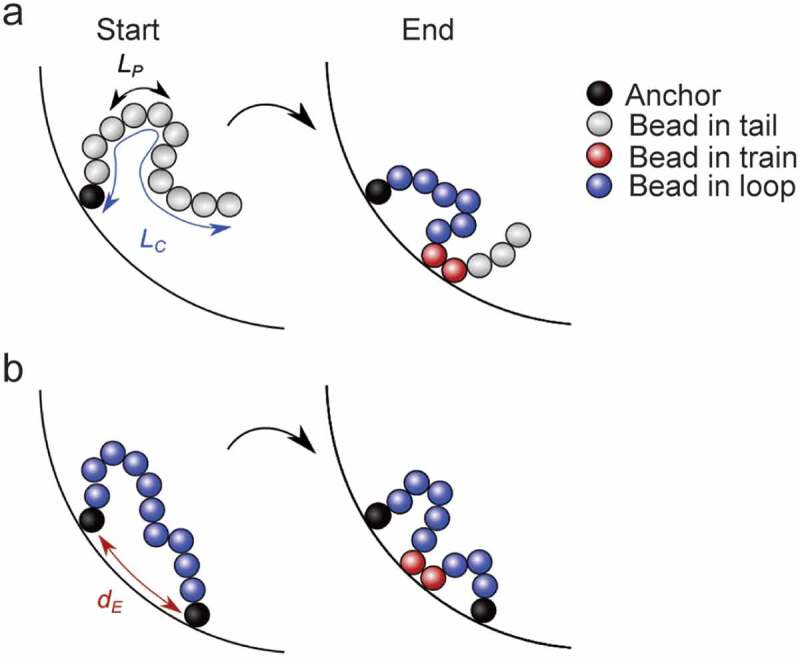
Modeling interactions of chromatin with the nuclear periphery (NP): polymer configurations and physical parameters examined. Chromatin is modeled as a polymer (or chain) of 12 beads pinned by (a) one anchor or (b) two anchors to a surface NP (curved surface) representing the nuclear lamina. The chromatin polymer is characterized by its contour length *L_C_* (here 360 nm), its persistence length *L_P_* defining its bending property (stiffness), and the Euclidian distance *d_E_* between the two anchors along NP. During Monte Carlo simulations, the polymer may change configuration (arrow) with respect to NP to adopt tail, train and/or loop configurations. Considering the genomic scale examined (~50 kb), we model chromatin behavior at the nuclear lamina at a sub-LAD scale with anchor points recapitulating chromatin interactions with lamins within a LAD

Our modeling is based on a self-avoiding polymer model [34] adapted to the genomic length scale of a sub-LAD segment at the nuclear lamina. We modeled chromatin as a chain of *N* = 12 connected hard beads of radius *r_bead_* = 15 nm (Figure 1a). Based on estimations of chromatin linear mass density (120–150 bp/nm) [35], genomic length *L_G_* of the modeled fiber is ~40-55 kb, which we set to 50 kb. This allows us to model chromatin behavior at the nuclear lamina at a sub-LAD scale, given a typical LAD size ranging from >100 kb to several megabases [3]. Note that genomic scale of our simulations may also model small LADs or interaction domains, in the tens of kb range, which have also been reported predominantly for A-type lamins [11,14,15,36]. This small system size also allows statistical sampling from a high number of simulations to enable detection of potentially tiny effects of the parameters tested. Polymer contour length is thus *L_C_ = 2r_bead_ (N-1)* = 360 nm, or ~1 Kuhn length (twice L_P_) of the most rigid polymer considered below.

## Configuration of chromatin chain anchoring to the nuclear periphery

In the model, the polymer is pinned to a hard surface NP at one end (bead 1) or both ends (bead 1, 12) of the polymer, with an imposed height *h* = *r_bead_* = 15 nm above NP. This enables modeling chromatin behavior near the nuclear lamina to which a segment is constrained by one or two anchors (Figure 1a,b). When simulations include two anchors, their relative position at NP is defined by the Euclidean distance *d_E_* between them (Figure 1b). Since we set *L_C_* to 360 nm, we probed *d_E_* = 50 nm (relaxed polymer), 100 nm, 200 nm and 300 nm (stretched polymer) to allow fluctuations of the chain in a broad range of stretching conditions. To pin the second end of the polymer to NP (bead 12), we adjusted the code as follows. Bead 12 (unlike bead 1) is not pinned to NP from the start of the simulations. Rather, it follows the same initialization step as all free beads in the chain, and pinning occurs by applying a full Hamiltonian (see below) between bead 12 and the position of the second anchor. Once bead 12 is pinned, Monte Carlo moves are no longer allowed for this bead but the free beads in the chain can move and reach any position relative to NP and the anchors (Figure 1b). Polymer motion is constrained by a sphere modeling a nucleus of radius *r_nucleus_* = 5 µm.

## Hamiltonian governing the system

Thermal fluctuations of the polymer depend on its persistence length $L_{P}=\kappa /k_{B}T$, which defines its bending properties, where κ is the bending modulus, $k_{B}$ the Boltzmann constant and T the temperature [37]. *L_P_* depends on DNA structure and chromatin compaction, with estimates ranging from ~50 nm for double-stranded DNA to >300 nm for more compact or heterogeneous structures [38–41] (**Supplementary information 1**). Given the heterogeneity of chromatin compaction in the nucleus [42], we examined *L_P_* = 5, 50, 100 and 200 nm to explore the influence of polymer stiffness on its interaction with NP. The torsional degree of freedom was omitted because it plays no direct role in the present work. Local stiffness and connectivity of the chain are ensured by a full Hamiltonian *H*, which consists of three terms governing the system:

(1) $$H=U_{Bending}+U_{Stretching}+U_{Self-Avoiding}$$

$U_{bending}$ is the bending energy expressed by introducing the angle between three successive beads. The bending energy term is:

(2) $$U_{Bending}\left(i-1,i,i+1\right)=\frac{L_{P}k_{B}T}{2r_{bead}}\sum \left(1-\frac{\vec{r_{i-1,i}}.\vec{r_{i,i+1}}}{r_{i-1,i}r_{i,i+1}}\right)$$

where $L_{P}k_{B}T/2r_{bead}$ controls the strength of the bending potential, $\vec{r_{i-1,i}}$ with *i* = 1, …,*N* is the vector connecting the center of bead *i*-1 and bead *i*, and $r_{i-1,i}$ is its norm.

$U_{Stretching}$ describes the potential energy corresponding to the stretching force applied to consecutive beads to maintain continuity of the chain [34]:

$U_{Stretching}\left(i,i+1\right)=\frac{L_{P}k_{B}T}{r_{bead}^{3}}\sum^{{\left(\vec{r_{i}}-\vec{r_{i+1}}-2r_{bead}\right)}^{2}}$ (3)

where $2r_{bead}$ is the equilibrium length of a chain segment (here, two bead radii).

$U_{Self-Avoiding}$ accounts for the excluded volume, and is the sum of two repulsive potentials: the purely repulsive Weeks-Chandler-Andersen potential $U_{LJ}$ [43], and the sum of interactions between beads and NP. The potential $U_{LJ}$ was designed to model excluded volume interactions by a short-range repulsive force between nonconsecutive beads. Thus, only the repulsive part of a truncated and shifted Lennard-Jones potential is taken into account:

(4) $$U_{LJ}\left(i,j\right)=\left\{\begin{array}{cc} 4\in \left[{\left(\frac{\sigma }{r_{i,j}}\right)}^{12}-2{\left(\frac{\sigma }{r_{i,j}}\right)}^{6}+\frac{1}{4}\right] & ,r_{i,j}\leq 2^{1/6}\sigma \\ 0 & ,r_{i,j}>2^{1/6}\sigma \end{array}\right.$$

here σ is the collision diameter with $2r_{bead}=2^{1/6}\sigma$, and ∈ is the depth of the potential with $\in =k_{B}T$. Equation 3 states that the center-to-center distance between any two beads cannot be less than the bead diameter. Lastly, the interaction between beads and NP is designed to retain any bead trying to escape the nucleus space by a restoring force modeled as a spring of stiffness $k_{B}T/r_{bead}^{2}$.

### Modeling polymer behavior near a surface fitted with an attraction potential

When relevant, we promoted polymer adsorption by conferring NP with an attraction potential toward the chromatin polymer. When a monomer is in the nucleus space, it experiences an isotropic osmotic pressure from its neighborhood. Close to NP, this pressure becomes anisotropic and the monomer experiences a net depletion force pushing it toward NP [44]. Its range is nanometric, defined by the typical molecular size in a crowded environment, and set here to the bead radius. This can be modeled as an attraction potential in the order of the thermal energy $k_{B}T$ [45]. We modeled this as a short-range 12–6 Lennard-Jones potential used in modeling chromatin–lamina interactions [32]. This was done using a modified 10–4 Lennard-Jones potential, obtained by integration of the 12–6 Lennard-Jones interactions over a spherical neutral surface [46]:

(5) $$U_{LJ}\left(i,j\right)=4\in \pi \frac{R_{c}}{r_{i}}\left(\frac{1}{5}\left[{\left(\frac{\sigma }{R_{c}-r_{i}}\right)}^{10}-{\left(\frac{\sigma }{R_{c}+r_{i}}\right)}^{10}\right]-\frac{\in_{ads}}{2}\left[{\left(\frac{\sigma }{R_{c}-r_{i}}\right)}^{4}-{\left(\frac{\sigma }{R_{c}+r_{i}}\right)}^{4}\right]\right)$$

where the attractive sphere radius is $R_{c}=r_{nucleus}-r_{bead}$, $r_{i}$ is the distance of the center of bead *i* to the nucleus center, $\in_{ads}$ is the attraction strength of the surface, set here to 0.005, 0.01, 0.05, 0.1 and 1; $\sigma =r_{bead}$ and $\in =1k_{B}T$.

## Kinetic Monte Carlo simulation

Out-of-equilibrium dynamics is explored through Kinetic Monte Carlo simulations. Energy variation at each step must satisfy $\Delta U\ll k_{B}T$ [47]. In a simulation, a Monte Carlo algorithm ensures movement of a free bead in the chain. A Metropolis criterion is applied, where the probability of accepting changes from a Monte Carlo Step $\left(MCStep\right)(i)$to $MCStep\left(i+1\right)$ equals 1 if energy in $MCStep\left(i+1\right)$ is lower than energy in $MCStep\left(i\right)$, and $exp\left(-\beta \left(E\left[MCStep\left(i+1\right)\right]-E\left[MCStep\left(i\right)\right]\right)\right)$ if not [48]. At each *MCStep*, a move *dr* is attempted for a random bead among the free beads and accepted if compatible with the energy requirement. This move is made by displacing a bead randomly within a sphere of radius *R_move_ = r_bead_/5* [34] centered on bead position. A Monte Carlo sweep (*MCSweep*) is a sequence of *MCStep*s where on average each bead moves once per *MCSweep*.

One simulation consists of 5 × 10^8^ *MCSweeps*, enabling sufficient space exploration by the polymer. We associated the duration of displacements for each *MCSweep* to a diffusion constant characterizing the motion of chromatin near NP extrapolated from a previous study [49] (10^−4^ µm^2^/s). Using this approximation, we calculated the maximal magnitude of displacement at each *MCSweep* to be *dr *= 3 nm and the duration of each *MCSweep* to be *dt *= 0.09 s. To initialize the simulation, each bead center is positioned 15 nm above NP, forming a straight chain. The pre-equilibration phase (5x10^6^ *MCSweeps*) is removed from downstream analyses. Resulting values are obtained from configuration sampling every 1000 *MCSweeps* and are an average (± s.e.m.) of three independent simulations.

## Data viewing

Plots were generated using Mathematica v11 under IRSAMC license No. 4730–0161.

## Results

### Experimental conditions

We addressed how chromatin-lamina interaction constraints impact chromatin conformation at a sub-LAD scale at a nuclear periphery NP (see **Figure 1**). We modeled a chromatin fiber of genomic length *L_G_* = 50 kb as a polymer of 12 connected beads (*i* = 1–12), of fixed contour length *L_C_* = 360 nm, and of stiffness increasing from a flexible chain (*L_P_* = 5 nm) to a near-rigid chain (*L_P_* = 200 nm). The chain contained one or two beads pinned to the surface NP (*i* = 1 or *i* = 1, 12) with increasing Euclidean distance between them (*d_E_* = 50–300 nm), resulting in a relaxed chain (d_E_ = 50 nm) or stretched chain (d_E_ = 300 nm). Given the range of chromatin persistence lengths reported in the literature for various degrees of DNA or chromatin compaction (**Supplementary information 1**), the data generated in this study for L_P_ = 5 nm (flexible chain) or 50 nm (semi-flexible chain) may be interpreted as the behavior of euchromatic domains interacting with lamins, either as narrow euchromatic LADs [11,14,15,36] or as micro-domains of lamin interaction within LADs [21]. Larger polymer persistence lengths (L_P_ = 100 or 200 nm) likely model the behavior of heterochromatin at the nuclear lamina, as found in *bona fide* LADs.

### Behavior of the chromatin chain anchored to a neutral surface

Distances between beads and NP

**Figure 2.**
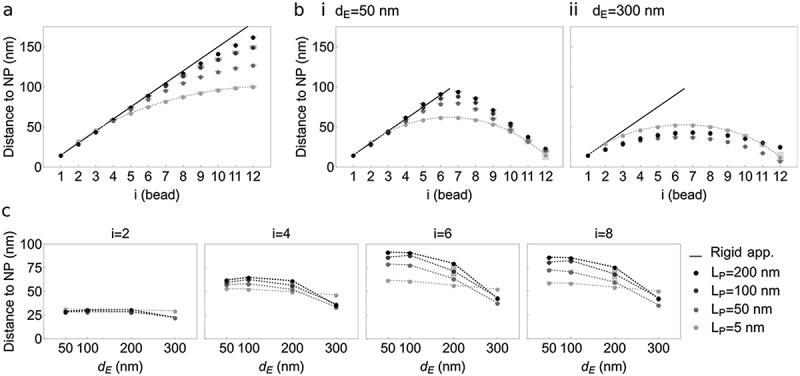
Behavior of a polymer pinned by one or two anchors to a surface NP without attraction potential. This experiment models the space explored (distance to NP) by a chromatin polymer anchored at one or both ends to a surface representing the nuclear lamina devoid of any attraction strength toward the polymer. (a) Mean distance from bead center to NP along the polymer, with bead 1 as the sole anchor. Gray shades represent increasing polymer stiffness *L_P_* (legend bottom right), from flexible (L_P_ = 5 nm) to near-rigid (L_P_ = 200 nm). (b) Mean distance from bead center to NP along a polymer anchored at both ends (beads 1 and 12) to NP, with a Euclidian distance between them of (i) *d_E_* = 50 nm (relaxed polymer) and (ii) *d_E_* = 300 nm (stretched polymer). Data for other *d_E_* values are shown in Figure S1a. In (a) and (b), black lines represent the theoretical rigid approximation (see Supplemental information 2); gray shades represent increasing *L_P_*; lines connect data points for *L_P_* = 5 nm for clearer visualization of the trends. (c) Distance from bead center to NP as a function of Euclidian distance *d_E_* between anchors, for increasing polymer stiffness *L_P_* (gray shades) and at indicated bead position *i* along the chain (top). Given approximations of L_P_ as a function of chromatin compaction (Supplemental information 1), our simulations model chromatin behavior at the nuclear periphery for euchromatic domains (L_P_ = 5 and 50 nm) and heterochromatic domains (L_P_ = 100 and 200 nm)

Considering NP as a surface with no attraction potential, we examined bead-NP distances as a function of bead position along the chain, chain stiffness, number of anchors and distance *d_E_* between them. Since our simulations occur within a rigid/semi-flexible system, we first established chain behavior with a theoretical rigid limit where *L_C_* ≪ *L_P_* (**Supplementary Information 2**; Figure 2a; black line). For one-anchor chains, increasing polymer stiffness leads to increasing bead-NP distances tending toward a rigid rod exploring the accessible space Figure 2a). With two anchors, bead-NP distance adopts a nearly symmetrical profile from the chain mid-point (Figure 2b**; Figure S1a**). With increasing rigidity, we detect more abrupt changes. Increasing *d_E_* lowers bead-NP distances for each bead as configurations become constrained near NP by stretching. Simulations also reflect a behavior transition between *d_E_* = 200 and 300 nm (Figure 2c). For *d_E_ *= 300 nm (stretched polymer), bead-NP distance is larger for a flexible chain (*L_P_* = 5 nm) than for semi-flexible chain (*L_P_* = 50 nm; Figure 2c). This suggests that between *d_E_* = 200 and 300 nm, the impact of *d_E_* dominates over that of *L_P_* in determining polymer proximity to NP.

These data indicate that as expected, a flexible chain can exhibit greater distances to NP than a more rigid polymer; however, for a relatively stretched polymer, stretching may override the impact of polymer stiffness on this behavior. We imply that a euchromatic domain may explore a greater space near the nuclear lamina than a more rigid heterochromatic domain – here assuming that no ‘force’ is exerted between chromatin and the lamina. If chromatin is stretched along the lamina, however, the impact of chromatin rigidity is minimal relative to its stretch.

Tail-train-loop configurations at NP

Polymer configurations near NP can be described as sequences of tails, trains and/or loops (see **Figure 1**). A tail represents a fully desorbed segment with one anchored end. A train represents one anchored bead or a sequence of consecutive beads adsorbed to NP with a distance from bead center to NP surface ≤30 nm (one bead radius plus a 15-nm adsorption zone from NP). A loop consists of contiguous desorbed beads separated by two trains (or anchors). At each iteration, multiple polymer segments can be in train or loop configurations, but at equilibrium, only one or zero tail can by definition be found in chains with one or two anchors, respectively. To characterize polymer configurations near NP, we analyzed the propensity of each bead along the chain to be in a tail, train or loop configuration.

**Figure 3.**
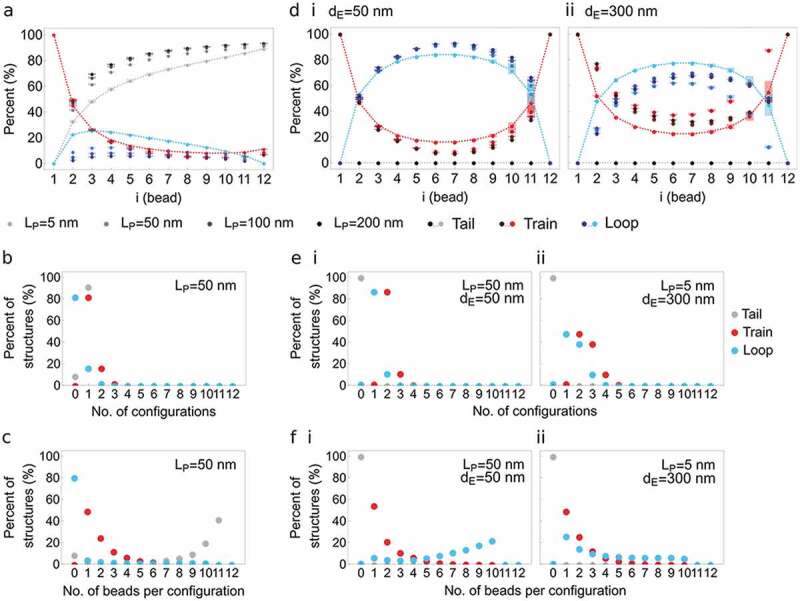
Tail-train-loop configurations of a chromatin polymer pinned at one or both ends to a neutral surface NP. This experiment models the conformations of a chromatin polymer anchored at one or both ends to a nuclear lamina devoid of attraction strength, at the end of simulations. (a) Percentage of structures with a tail (gray), train (red) and loop (blue) configuration as a function of bead position along the chain (x axis), with one anchor to NP (bead 1). Increasing color intensity depicts increasing polymer stiffness *L_P_* (legend). Train configurations being insensitive to *L_P_*, the red symbols appear superimposed. Lines are shown for data points at *L_P_* = 5 nm (modeling a flexible chromatin chain) for easier visualization of the trends. (b,c) Percentage of one-anchor point structures with (b) the indicated number of tails, trains and loops (x axis) and (c) the indicated number of beads in each of these tail (gray), train (red) and loop (blue) configurations. (d) Percentage of structures with a tail, train, loop configuration for a polymer pinned at both ends to NP (bead 1, 12), as a function of polymer stiffness L_P_ (color intensity; legend). Data are shown separately for two Euclidian distances along NP: (i) *d_E_* = 50 nm (relaxed chain) and (ii) *d_E_* = 300 nm (stretched chain). Data for other *d_E_* values are shown in Figure S1b. Lines connect data points for *L_P_* = 5 nm (flexible chain) for clearer visualization of the trends. (e,f) Percentage of two-anchor point structures with (e) the indicated number of configuration and (e) the indicated number of beads in each configuration as in (b) and (c); shown for two examples of *L_P_* (a flexible chain with L_P_ = 5 nm and a semi-flexible chain with L_P_ = 50 nm), and two *d_E_* conditions (a relaxed chain with d_E_ = 50 nm and a stretched chain with d_E_ = 300 nm). Impact of polymer flexibility dominates over polymer stretching on its configurations at NP. We infer that chromatin stiffness influences contacts with the lamina to a greater extent than how stretched the chromatin domain is: greater flexibility, such as in euchromatin relative to heterochromatin, favors multiple interaction configurations at the lamina

In simulations with one anchor (Figure 3a), the frequency of being in tail increases along the chain and tail is the dominant configuration irrespective of *L_P_*. Further, the highest frequencies of being in train occur near the anchor (bead 2–3) and train frequency decreases along the polymer independently of *L_P_*. Lastly, loop frequency is highest near the anchor, highest for a flexible chain (*L_P_* = 5 nm) and decreases with increasing chain stiffness in favor of tails. Thus with one anchor, the main geometry of the polymer is a tail, or a desorbed segment away from the interaction surface.

We next examined the number of tail-train-loop configurations and the number of beads in each configuration, i.e. the size of these configurations. For a semi-flexible chain (*L_P_* = 50 nm), most structures adopt a configuration of 0 loop, 1 train and 1 tail (Figure 3b), with the train mostly containing 1 or 2 beads (Figure 3c). This short train-long tail configuration also prevails with increasing polymer stiffness (**Figure S1b,c**). However, along a more flexible chain (*L_P_* = 5 nm), while 60% of the structures adopt a short train-long tail configuration, ~30% display 1 loop and 2 trains (**Figure S1b**), each mainly containing one or two beads (**Figure S1c**). This reveals a low frequency of transitions between train-tail and train-loop configurations in the most flexible polymers across simulations.

With two anchors, the only allowed tail-train-loop configurations are trains and loops (**Figure 3di,ii; Figure S1d**). Beads contiguous with the anchors display the most variable tail-train-loop configurations, with a 50% train or loop frequency. Progression away from the anchors along the chain is accompanied by a decreasing propensity to be in a train, and train frequency in the middle of the chain decreases with increasing polymer stiffness *L_p_* and distance *d_E_* between the anchors (**Figure 3di,ii; Figure S1d**). Interestingly, a stretched polymer (*d_E_* = 300 nm), especially when semi-flexible (*L_P_* = 50 nm), shows increased fluctuations between loops and trains (**Figure 3dii**). Thus, a polymer with two anchors favors loops over trains, and stiffness and stretching influence these configurations, further promoting loops.

Examination of the number of trains and loops along the chain and of the number of beads involved in these configurations shows that, for semi-flexible or rigid polymers (*L_P_* = 50–200 nm), with close or distant anchors, the main structure consists of 2 trains of 2 beads (the anchor and the adjacent bead) and 1 loop of 7–10 beads for *d_E_* ≤ 200 nm (**Figure 3ei,3fi; Figure S2**), or fewer for stretched polymers with *d_E_* = 300 nm (**Figure 3eii,3fii; Figure S2g,h**). With flexible polymers (*L_P_* = 5 nm), we note a higher frequency of 3 trains – 2 loops configurations (**Figure 3eii; Figure S2a,c,e,g**). The number of beads in loops is also more homogeneously distributed along the chain (**Figure 3fii**) regardless of stretching (**Figure S2b,d,f,h)**. These results indicate a dominant impact of polymer flexibility over stretching on its configuration at NP.

We may infer from these findings that stiffness a chromatin domain near the nuclear lamina influences its contacts with the lamina to a greater extent than how stretched the domain is: greater chromatin flexibility, such as of euchromatic relative to heterochromatin, favors multiple configurations at the lamina.

### Polymer behavior near a surface fitted with an attraction potential

#### Definition of adsorption and desorption regimes

Since no stable polymer interaction with NP occurred in our previous simulations, we promoted polymer adsorption to NP by providing NP with an attraction potential. This was motivated by observations that, while random encounters of chromatin with the lamina may occur in the nucleus, chromatin–lamin interactions are mediated by protein factors and/or chromatin states [4,9,29,50,51]. When a polymer experiences a short-range attraction by a wall, it can be adsorbed according to an attraction strength *ε_ads_* [52]. We defined a polymer adsorption behavior by the proportion of the polymer interacting with NP; this results from a competition between attraction potential which favors adsorption, and entropic repulsion which favors a desorbed state. We characterized adsorption/desorption transitions as a function of *ε_ads_*.

Bead trajectories as distances to NP in a chain with one anchor show that displacement is strongly influenced by attraction potential in a stiffness (*L_P_*)-dependent manner (**Figure S3**; shown for bead *i* = 6). Density of adsorbed states increases with increasing *ε_ads_*, and as expected a strong attraction potential abolishes polymer fluctuations.

**Figure 4.**
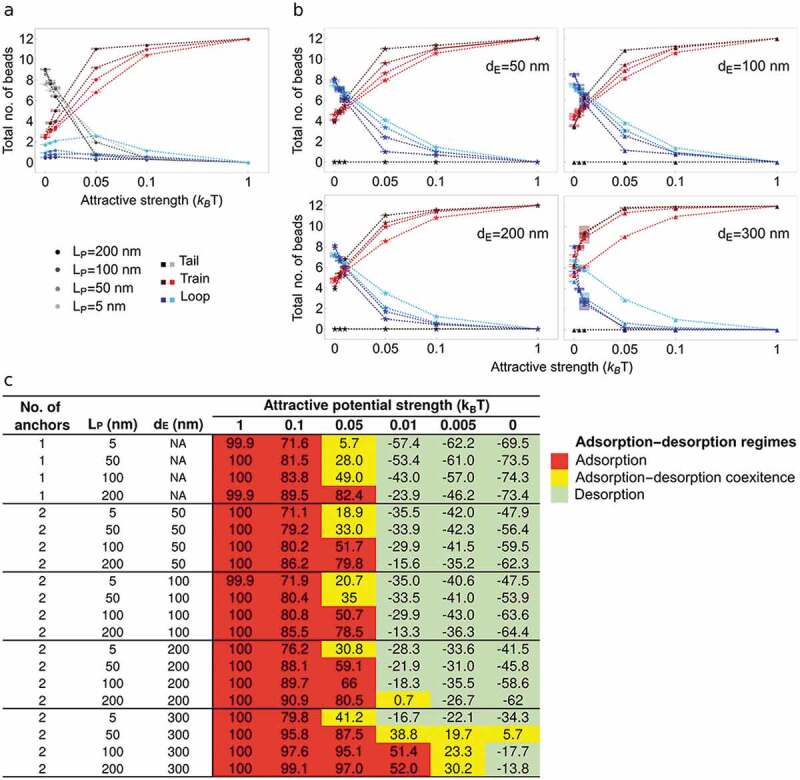
Polymer configurations at a surface NP fitted with an attraction potential toward the chromatin polymer. This experiment tests the effect of introducing a variable attraction force in the nuclear lamina on the conformation of chromatin at the lamina (in the form of tail, trains or loops) and on the size of these configurations (number of polymer beads). (a,b) Number of beads in tail-train-loop configurations as a function of attraction strength (x axis) and polymer stiffness (L_P_; gray, red and blue shades; legend) in simulations with (a) one anchor point and (b) two anchor points and indicated *d_E_* between them. (c) Definition of polymer adsorption/desorption regimes at NP from the proportion of beads in a tail-train-loop configuration (F_tail/train/loop_ = <F_train_> – <F_other_>), with: adsorption when F_tail/train/loop_ > 50% (red), adsorption-desorption transitions when 0 < F_tail/train/loop_ ≤ 50% (yellow), and desorption when F_tail/train/loop_ ≤ 0 (green). Numbers are F_tail/train/loop_ in percent of the modeled structures. We refer to Supplementary information 1 to relate chromatin polymer stiffness (L_P_) to chromatin compaction in the nucleus. While low persistence length (L_P_ = 5–50 nm; (semi)-flexible polymer) characterizes euchromatin, elevated persistence lengths (L_P_ = 100–200 nm; semi-flexible to rigid polymer) are properties of heterochromatin. We infer that regardless of the number of anchors, euchromatin is more prone than heterochromatin to stochastic associations with the lamina (modeled as adsorption-desorption regimes) even when it is stretched

We next determined the total number of beads in tail-train-loop configurations as a function of attraction potential. A potential promotes a train configuration at the expense of tails or loops (Figure 4a**,b**); this is enhanced with increasing polymer stiffness (Figure 4a**,b**, red lines). Further stretching the polymer exacerbates this behavior (Figure 4b). From this information, we defined adsorption/desorption regimes by calculating the comparative frequency *F* of being in a given tail-train-loop configuration, as:

(5) $$F_{tail/train/loop}=\left\langle F_{train}\right\rangle -\left\langle F_{other}\right\rangle$$

where *F_train_* is the frequency of all free beads to be in a train, and *F_other_* is the frequency of being in other configurations, i.e. (*F_loop_* + *F_tail_*) for 1 anchor, and *F_loop_* for 2 anchors. We arbitrarily defined three adsorption/desorption regimes as adsorption when *F_tail/train/loop_* > 50%, adsorption-desorption when 0 < *F_tail/train/loop_* ≤ 50%, and desorption when *F_tail/train/loop_* ≤ 0. Results are summarized in Figure 4c.

#### Characterization of adsorption and desorption regimes

**Figure 5.**
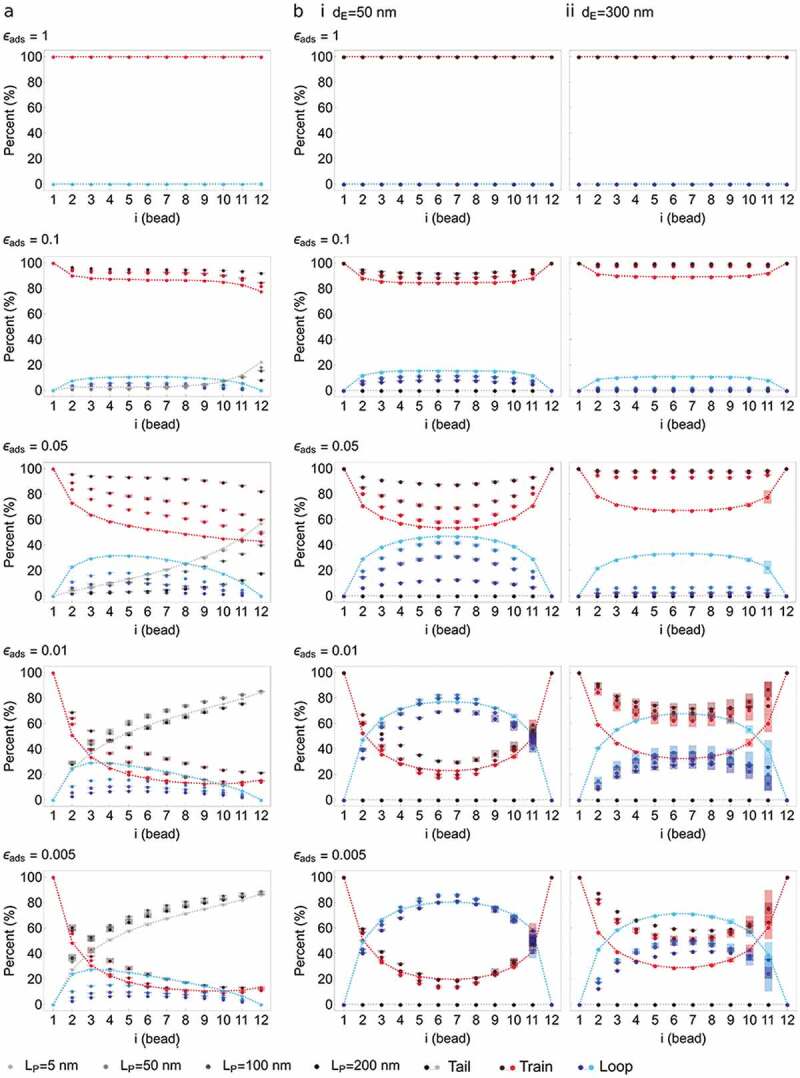
Tail-train-loop configurations at a surface NP fitted with varying attraction potentials. This experiment determines the conformations of a chromatin chain (tail, trains and loops) anchored at one or both ends to the lamina which here is fitted with a variable attraction potential (*ε_ads_*). A strong potential (*ε_ads_* = 1; top graphs) stably attracts the whole chain to the surface, while a very weak potential (*ε_ads_* = 0.005; bottom graphs) exerts a minimal, if any, effect. (a) Percentage of beads in tail-train-loop configurations (legend) in simulations with one anchor point, as a function of bead position along the chain (x axis), attraction potential (ε_ads_ = 0.005–1) and chain stiffness *L_P_* (legend). (b) Same as in (a) for a polymer with two anchor points and as a function of *d_E_*; (i) *d_E_* = 50 nm; (ii) *d_E_* = 300 nm. Lines connect data points for *L_P_* = 5 nm for clearer visualization of the trends (tail, train, loop). The data demonstrate the requirement for an attraction potential to generate long-lasting interactions with NP, and that these involve cooperative recruitment of neighboring beads. Propensity for cooperative recruitment is exacerbated by polymer stiffness and stretching. Implications are that, at the genomic scale examined here, recruitment of chromatin to the nuclear lamina invokes extensions of existing LADs (or of lamina-bound sites within LADs). This recruitment is enhanced for heterochromatic domains such as those found in *bona fide* LADs

Based on the above classification, we find that simulations with *ε_ads_* = 1 elicit a strong adsorption regime regardless of the number of anchors, polymer stiffness *L_P_* and distance *d_E_* between anchors (Figure 4c**; Figure S4a,b**) in all structures (Figure 5a**,b; Figure S5a,b**). *ε_ads_* = 0.1 also induces strong adsorption (Figure 4c) but enables tiny fluctuations between trains and loops regardless of the number of anchors, *L_P_* and *d_E_* (Figure 5a**,b; Figure S4a,b; Figure S5a,b**). *ε_ads_* = 0.05 also promotes adsorption, particularly in more rigid and stretched polymers (Figure 4c, red cells; Figure 5a**,b; Figure S5a,b**). Under these conditions, adsorption limits looping and dissociation from NP is essentially suppressed. *ε_ads_* = 0.05 elicits adsorption–desorption transitions in flexible and semi-rigid polymers (L_P_ 5–50 nm) (Figure 4c, yellow cells), with configurations oscillating between trains and loops (Figure 5a**,b; Figure S4a,b; Figure S5a,b**). Greater chain flexibility increases the propensity of forming multiple trains with no preferential position along the chain (**Figure S6a,b**; compare, e.g. *L_P_* = 5 and 100 nm).

Lowering the attraction potential to *ε_ads_* = 0.01 promotes adsorption or adsorption–desorption transition regimes for the most rigid or stretched polymers (Figure 4c, yellow cells), yet most reveal complete chain desorption irrespective of polymer properties (green cells). Further lowering *ε_ads_* to 0.005 leads to desorption when the chain is non-flexible (*L_P_* > 5 nm) and stretched (d_E_ = 300 nm; Figure 4c, yellow cells; Figure 5a**,b; Figure S4a,b; Figure S5a,b**). With two anchors, increasing polymer flexibility also favors multiple trains (**Figure S6c,d**; compare *L_P_* = 5 and 100 nm; **Figure S6e,f**). Below adsorption–desorption transition regimes, loops merge with other loops or with a tail due to the release of adsorbed segments (trains) from the now non-attractive surface. Lastly, with two anchors, increasing the distance *d_E_* between them favors adsorption even in a flexible polymer with *ε_ads_* as low as 0.01 (see Figure 4c).

We infer from these findings that regardless of the number of anchors to the lamina, a flexible euchromatic domain is more prone than a more rigid heterochromatic domain to undergo stochastic associations (modeled as adsorption-desorption regimes) even when it is stretched along the lamina.

## Discussion

We have examined to what extent the physical properties of a polymer modeling a chromatin segment at kilobase scale influence interactions with a surface NP representing the nuclear lamina. We show a dominant effect of polymer stiffness over stretching on its mode of interaction with NP, and infer from our models that a flexible euchromatin domain exhibits more stochastic interactions with the lamina than stiffer heterochromatin domains characterizing cLADs. We show that conferring an attraction potential to NP [53] is necessary to elicit long-lasting interactions, in line with modeling predictions of chromatin-lamina associations at much larger genomic scales [30–33]. Tuning this potential promotes full adsorption of the chain, complete desorption, or adsorption-desorption regimes manifested by fluctuations between train and loop configurations along the chain.

### Physical considerations of our models

In a desorption regime, loops merge with other loops or with a tail as interacting domains (trains) are released from the surface. A simple scaling argument can account for this [52]. When the polymer is adsorbed, energy gain is in the order of $N\in_{ads}k_{B}T$. For a polymer with pinned ends, entropic cost of adsorption is only caused by the loss of internal degrees of freedom as there is no translational entropy when the polymer is desorbed. Entropy loss is in the order of $N_{K}k_{B}T$, where $N_{K}=L_{C}/\left(2L_{P}\right)$ is the number of Kuhn segments in the chain. In a rigid polymer (*L_C_* ∼ x≺ *L_P_*), this entropic cost is in the order of $k_{B}T$. Under these conditions, adsorption is favored when ∈ads∼ x≻1/N, which sets a lower *ε_ads_* threshold leading to adsorption. With two anchors, increasing the Euclidian distance *d_E_* between them favors adsorption even for a flexible polymer and with a weak attraction potential (≪$k_{B}T$ per bead). This can again be explained by entropy reduction under adsorption. When *d_E_* is close to the contour length *L_C_*, the chain is stretched and when desorbed, its fully accessible fluctuation modes consist of several short-range adsorbed and desorbed segments. There, entropy loss upon adsorption is in the order of a few $k_{B}T$ even for a flexible chain. Without attraction potential, no significant enhancement in polymer–surface interactions occurs upon stretching, confirming the central role of an attraction force, even weak, in eliciting long-lasting interactions.

We have limited our simulations to 12-bead chains to enable frequent statistical sampling with high precision. Examining longer chains would however be useful to model entire LADs. One may speculate on features of such models, considering a 2-anchor and non-stretched polymer (stretching is not the most critical parameter governing polymer behavior at NP; this study) with L_P_ ~100 nm (a typical value for chromatin) and of contour length L_C_ ≫ L_P_ and d_E_. We anticipate that cooperative contacts with the lamina close to the anchors would similarly occur, while mid-chain contacts, reflecting chromatin–lamina interactions far away from anchors, would be more frequent. For non-stretched chains, mid-chain contact features are expected to be independent of chain length provided that it is longer than L_P_. Of note, our simulations with L_P_ = 100 nm, d_E_ = 50 nm and ε_ads_ = 0.05 or 0.1 (**Figure S6e,f**) show that the most frequent outcomes are simultaneous adsorption of all beads, or 1 loop and 2 trains of typically ≤ 6 beads. Trains cannot be much shorter than L_P_ as this would require strong bending of the chain, which is energetically unfavorable. In addition, we do not expect trains to be longer in longer chains because trains result from local cooperative bead adsorption non-correlated over long distances. Similarly, in the range of our parameters, typical loop size is ≤6 beads, which is much shorter than chain length; thus, we anticipate that increasing chain length would not significantly affect this loop size. However, in longer chains, we could expect several loops ‘sliding’ between the anchors, with several mid-chain contacts (see also below).

### Biological implications of our models

Without attraction potential, only short-lived random polymer encounters with NP occur, while interactions can be modulated by tuning a potential. Our results recapitulate observations that variable chromatin-lamin contacts occur between cells in a population [17]. In line with our models, more stable interactions, such as in cLADs, require proteins in the inner nuclear membrane, the nuclear lamina or chromatin, such as LBR, LEM-domain proteins, lamin A/C [29] or barrier-to-autointegration factor [54]. The attraction potential conferred to our models globally represents the range of factors required to address or anchor loci to the nuclear envelope [4,5,9,51,55–57].

Single-cell analysis of lamin B1–chromatin interaction reveals significant cell-to-cell variation in LADs [17]. Chromatin-lamin contact frequencies vary across the genome, being lowest at vLADs and in gene-rich regions, a hallmark of euchromatin, and highest in heterochromatic and gene-poor LADs [17]. The single-cell data are consistent with our modeling outcomes: these highlight the greater propensity of a flexible/semi-flexible chain (modeling euchromatin) to display dynamic adsorption–desorption transitions with a ‘lamina’ and to interact at multiple and non-preferred positions along the chain, than a rigid polymer. In contrast, under similar adsorption-desorption regimes, a more rigid polymer modeling heterochromatin is more prone to undergo long-lasting interactions involving larger domains. Stochasticity in configurations of flexible chromatin polymer models at the lamina therefore concords with the variability of lamin interactions in euchromatic parts of the genome [8,10,11,36,58]. Supporting our polymer models, restrain-based 3D genome models [18,19] also predict more variegated positioning of euchromatic domains at the nuclear periphery across models, recapitulating the cell-to-cell variability in lamin-chromatin contacts.

Our data argue that the interaction of a chromatin polymer with the nuclear lamina is seeded from the anchors and invokes cooperative recruitment of neighboring beads. In a nucleus context, our models are supported by observations that most of the variability of vLADs invokes extensions of already existing LADs [9–11,58,59] or lamina-bound chromatin regions within LADs, rather than binding of domains distant from existing anchors. Our models infer that this cooperative recruitment is enhanced with more rigid heterochromatin domains such as those in *bona fide* LADs; it is also favored by polymer stretching, altogether demonstrating an interplay between attraction potential, persistence length and distance between anchors. This suggests that the physical properties of chromatin at the tens of kb scale influence the parameters required for association with the nuclear lamina. Supporting this view, local transcriptional and epigenetic environments [13,15] modulate chromatin contacts with lamins.

Euchromatin, which has lower L_P_ than heterochromatin [35] and is therefore more flexible, can also interact with lamins [11,13–15]. Our simulations show that a polymer of low persistence length displays more structural variability at NP than a more rigid polymer modeling a heterochromatin domain. This is manifested in our simulations by enhanced fluctuations between train and loop configurations. Similarly, the smaller L_P_, the greater the propensity to form multiple trains. These results concur with observations that lamin interactions with euchromatin are more variable than heterochromatic LADs [11,14,15].

**Figure 6.**
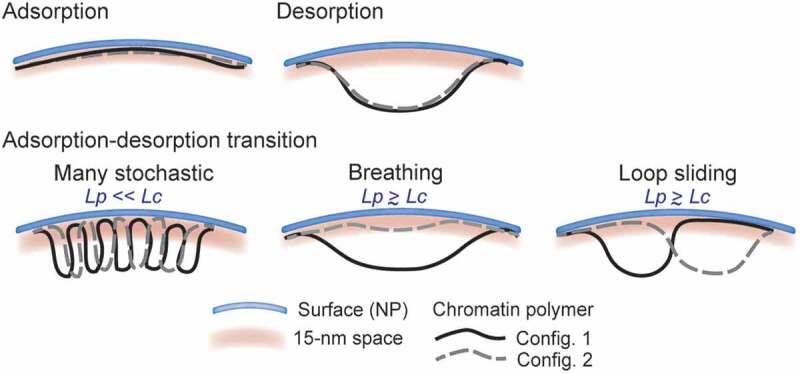
Interpretations of polymer simulations on chromatin configurations at the nuclear lamina. The genomic scale of our models (~50 kb) predicts chromatin behavior at the lamina within LADs rather than the behavior of entire LADs. We note however that simulations may also model narrow LADs of < 100 kb [11,14,15,36]. Monte Carlo simulations of the behavior of chromatin anchored at both ends to the lamina reveal various configurations depending on the ‘attraction strength’ of the system. A strong attraction potential induces a strong adsorption regime where the entire chromatin segment fully interacts with the lamina as a single ‘train’ (Adsorption). The absence of, or a very weak, attraction strength results in a desorbed chromatin segment which is only pined to the lamina by the preexisting anchors (Desorption). Within a LAD, such desorbed regions model a micro-loop domain of chromatin not bound to lamins, a configuration which has been as proposed earlier [21] and supported by the genomic and biochemical heterogeneity of LADs [20]. Between these adsorption and desorption extremes, weak but significant attraction potentials result in chromatin adsorption-desorption transition regimes. These can be interpreted as (i) many short-lived stochastic chromatin interactions with the lamina at any position along the segment (left); here, L_P_ is greatly inferior to the chromatin contour length L_C_, which characterizes euchromatin. (ii) Lamina contacts and releases of an entire chromatin segment occur (except at the anchors), yielding a ‘breathing’ pattern (middle). (iii) Lamina contacts with and releases of sub-domains of relatively constant size but at varying positions occur along the segment, hence a ‘sliding loop’ pattern (right). The latter patterns are observed with L_P_ approximating or greater than L_C_, modeling the behavior of a typical heterochromatic LAD sub-domain

Our study at kilobase scale raises the issue of whether a LAD consists of a domain, which wholly interacts with the lamina or rather consists of smaller domains that associate/dissociate with/from the lamina within a LAD [3]. Under conditions of adsorption-desorption regimes (fluctuations between trains and loops), our data are compatible with several models of chromatin–lamin interactions (**Figure 6**). (i) Many stochastic interactions can occur along a given polymer segment; this is only possible for flexible polymers (*L_P_* ≪ *L_C_*) and is supported by simulations revealing two (as opposed to one) prevailing configurations (2 trains-1 loop and 3 trains-2 loops). In a chromatin context, our simulation may therefore model lamin interactions with euchromatic sub-LAD domains [20], punctual sites of phosphorylated lamin A interaction with enhancers [15], dynamic euchromatic lamin A/C-chromatin interactions [11,36], transient euchromatin interactions with lamin B1 during the circadian cycle [58], or interactions of lamin B1 with gene-rich euchromatic regions in senescent cells [27]. (ii) Repeated adsorption/desorption transitions can occur along the chain, giving rise to a polymer ‘breathing’ pattern; this view emerges from one dominant configuration (2 trains-1 loop) in simulations with stretched and semi-flexible to rigid polymers. The number of beads in these configurations is homogeneously distributed along the chain, demonstrating variability in the length of these configurations. This could plausibly model rhythmic associations of chromatin with the lamina [58]. (iii) Lastly, a loop can form at various positions along the chain, yielding 2 trains-1 loop configurations of essentially constant lengths, suggesting a ‘loop sliding’ pattern between the anchors; this is also valid for a stretched or near rigid polymer (*L_P_* ∼ x≻ *L_C_*). As discussed earlier, in the range the parameters explored in this study, we speculate that increasing chain length would not significantly affect the maximum loop size observed in simulations; however, in longer chains, we anticipate several loops ‘sliding’ between the anchors, with several mid-chain contacts. Altogether, our modeling data predict that transitions in chromatin configurations at the nuclear lamina are modulated by an interplay between an ‘attraction strength’ with the nuclear envelope, chromatin stiffness and Euclidian distance between existing chromatin anchors.

## Supplementary Material

Supplemental Material

## References

[1] Buchwalter A, Kaneshiro JM, Hetzer MW. Coaching from the sidelines: the nuclear periphery in genome regulation. Nat Rev Genet. 2019;20(1):39–50. 30356165 10.1038/s41576-018-0063-5 PMC6355253

[2] Burke B, Stewart CL. The nuclear lamins: flexibility in function. Nat Rev Mol Cell Biol. 2013;14(1):13–24. 23212477 10.1038/nrm3488

[3] Briand N, Collas P. Lamina-associated domains: peripheral matters and internal affairs. Genome Biol. 2020;21(1):85. 32241294 10.1186/s13059-020-02003-5 PMC7114793

[4] Czapiewski R, Robson MI, Schirmer EC. Anchoring a leviathan: how the nuclear membrane tethers the genome. Front Genet. 2016;7:82. 27200088 10.3389/fgene.2016.00082 PMC4859327

[5] Zuleger N, Boyle S, Kelly DA, *et al*. Specific nuclear envelope transmembrane proteins can promote the location of chromosomes to and from the nuclear periphery. Genome Biol. 2013;14(2):R14. 23414781 10.1186/gb-2013-14-2-r14 PMC4053941

[6] Worman HJ, Schirmer EC. Nuclear membrane diversity: underlying tissue-specific pathologies in disease? Curr Opin Cell Biol. 2015;34:101–112. 26115475 10.1016/j.ceb.2015.06.003 PMC4522394

[7] Kim Y, Zheng X, Zheng Y. Role of lamins in 3D genome organization and global gene expression. Nucleus. 2019;10(1):33–41. 30755082 10.1080/19491034.2019.1578601 PMC6380387

[8] Meuleman W, Peric-Hupkes D, Kind J, et al. Constitutive nuclear lamina-genome interactions are highly conserved and associated with A/T-rich sequence. Genome Res. 2013;23:270–280. 23124521 10.1101/gr.141028.112 PMC3561868

[9] Harr JC, Luperchio TR, Wong X, et al. Directed targeting of chromatin to the nuclear lamina is mediated by chromatin state and A-type lamins. J Cell Biol. 2015;208(1):33–52. 25559185 10.1083/jcb.201405110 PMC4284222

[10] Peric-Hupkes D, Meuleman W, Pagie L, *et al*. Molecular maps of the reorganization of genome-nuclear lamina interactions during differentiation. Mol Cell. 2010;38:603–613. 20513434 10.1016/j.molcel.2010.03.016 PMC5975946

[11] Rønningen T, Shah A, Oldenburg AR, et al. Prepatterning of differentiation-driven nuclear lamin A/C-associated chromatin domains by GlcNAcylated histone H2B. Genome Res. 2015;25(12):1825–1835. 26359231 10.1101/gr.193748.115 PMC4665004

[12] Robson MI, de Las Heras JI, Czapiewski R, et al. Tissue-specific gene repositioning by muscle nuclear membrane proteins enhances repression of critical developmental genes during myogenesis. Mol Cell. 2016;62(6):834–847. 27264872 10.1016/j.molcel.2016.04.035 PMC4914829

[13] Lund E, Oldenburg A, Delbarre E, et al. Lamin A/C-promoter interactions specify chromatin state-dependent transcription outcomes. Genome Res. 2013;23(10):1580–1589. 23861385 10.1101/gr.159400.113 PMC3787256

[14] Gesson K, Rescheneder P, Skoruppa MP, et al. A-type lamins bind both hetero- and euchromatin, the latter being regulated by lamina-associated polypeptide 2 alpha. Genome Res. 2016;26(4):462–473. 26798136 10.1101/gr.196220.115 PMC4817770

[15] Ikegami K, Secchia S, Almakki O, et al. Phosphorylated lamin a/c in the nuclear interior binds active enhancers associated with abnormal transcription in progeria. Dev Cell. 2020;52(699–713):e611. 10.1016/j.devcel.2020.02.011 PMC7201903 32208162

[16] Kind J, Pagie L, Ortabozkoyun H, et al. Single-cell dynamics of genome-nuclear lamina interactions. Cell. 2013;153(1):178–192. 23523135 10.1016/j.cell.2013.02.028

[17] Kind J, Pagie L, de Vries SS, *et al*. Genome-wide maps of nuclear lamina interactions in single human cells. Cell. 2015;163(1):134–147. 26365489 10.1016/j.cell.2015.08.040 PMC4583798

[18] Paulsen J, Sekelja M, Oldenburg AR, *et al*. Chrom3D: three-dimensional genome modeling from Hi-C and nuclear lamin-genome contacts. Genome Biol. 2017;18(1):21. 28137286 10.1186/s13059-016-1146-2 PMC5278575

[19] Li Q, Tjong H, Li X, et al. The three-dimensional genome organization of Drosophila melanogaster through data integration. Genome Biology. 2017;18(1):145. 28760140 10.1186/s13059-017-1264-5 PMC5576134

[20] Leemans C, van der Zwalm MCH, Brueckner L, et al. Promoter-intrinsic and local chromatin features determine gene repression in LADs. Cell. 2019;177(852–864):e814. 10.1016/j.cell.2019.03.009 PMC6506275 30982597

[21] Ikegami K, Egelhofer TA, Strome S, et al. Caenorhabditis elegans chromosome arms are anchored to the nuclear membrane via discontinuous association with LEM-2. Genome Biol. 2010;11(12):R120. 21176223 10.1186/gb-2010-11-12-r120 PMC3046480

[22] Lund EG, Oldenburg AR, Collas P. Enriched Domain Detector: a program for detection of wide genomic enrichment domains robust against local variations. Nucleic Acids Res. 2014;42(11):e92. 24782521 10.1093/nar/gku324 PMC4066758

[23] Paulsen J, Liyakat Ali TM, Nekrasov M, et al. Long-range interactions between topologically associating domains shape the four-dimensional genome during differentiation. Nat Genet. 2019;51(5):835–843. 31011212 10.1038/s41588-019-0392-0

[24] Narita M, Nunez S, Heard E, et al. Rb-mediated heterochromatin formation and silencing of E2F target genes during cellular senescence. Cell. 2003;113(6):703–716. 12809602 10.1016/s0092-8674(03)00401-x

[25] Chandra T, Ewels PA, Schoenfelder S, et al. Global reorganization of the nuclear landscape in senescent cells. Cell Rep. 2015;10(4):471–483. 25640177 10.1016/j.celrep.2014.12.055 PMC4542308

[26] Chandra T, Kirschner K, Thuret JY, *et al*. Independence of repressive histone marks and chromatin compaction during senescent heterochromatic layer formation. Mol Cell. 2012;47(2):203–214. 22795131 10.1016/j.molcel.2012.06.010 PMC3701408

[27] Sadaie M, Salama R, Carroll T, *et al*. Redistribution of the Lamin B1 genomic binding profile affects rearrangement of heterochromatic domains and SAHF formation during senescence. Genes Dev. 2013;27(16):1800–1808. 23964094 10.1101/gad.217281.113 PMC3759696

[28] Shah PP, Donahue G, Otte GL, *et al*. Lamin B1 depletion in senescent cells triggers large-scale changes in gene expression and the chromatin landscape. Genes Dev. 2013;27(16):1787–1799. 23934658 10.1101/gad.223834.113 PMC3759695

[29] Solovei I, Wang AS, Thanisch K, *et al*. LBR and lamin A/C sequentially tether peripheral heterochromatin and inversely regulate differentiation. Cell. 2013;152(3):584–598. 23374351 10.1016/j.cell.2013.01.009

[30] Ulianov SV, Doronin SA, Khrameeva EE, *et al*. Nuclear lamina integrity is required for proper spatial organization of chromatin in Drosophila. Nat Commun. 2019;10(1):1176. 30862957 10.1038/s41467-019-09185-y PMC6414625

[31] Falk M, Feodorova Y, Naumova N, *et al*. Heterochromatin drives compartmentalization of inverted and conventional nuclei. Nature. 2019;570(7761):395–399. 31168090 10.1038/s41586-019-1275-3 PMC7206897

[32] Chiang M, Michieletto D, Brackley CA, et al. Polymer modeling predicts chromosome reorganization in senescence. Cell Rep. 2019;28(3212–3223):e3216. 10.1016/j.celrep.2019.08.045 PMC6859504 31533042

[33] Sati S, Bonev B, Szabo Q, *et al*. 4D Genome rewiring during oncogene-induced and replicative senescence. Mol Cell. 2020;78(3):e529. 10.1016/j.molcel.2020.03.007 PMC7208559 32220303

[34] Manghi M, Tardin C, Baglio J, et al. Probing DNA conformational changes with high temporal resolution by tethered particle motion. Phys Biol. 2010;7(4):046003. 20952812 10.1088/1478-3975/7/4/046003

[35] Bystricky K, Heun P, Gehlen L, et al. Long-range compaction and flexibility of interphase chromatin in budding yeast analyzed by high-resolution imaging techniques. Proc Natl Acad Sci U S A. 2004;101(47):16495–16500. 15545610 10.1073/pnas.0402766101 PMC534505

[36] Forsberg F, Brunet A, Ali TML, et al. Interplay of lamin A and lamin B LADs on the radial positioning of chromatin. Nucleus. 2019;10(1):7–20. 30663495 10.1080/19491034.2019.1570810 PMC6363278

[37] Marko JF, Siggia ED. Stretching DNA. Macromolecules. 1995;28(26):8759–8770.

[38] Brunet A, Tardin C, Salome L, et al. Dependence of DNA persistence length on ionic strength of solutions with monovalent and divalent salts: a joint theory-experiment study. Macromolecules. 2015;48(11):3641–3652.

[39] Arbona JM, Herbert S, Fabre E, et al. Inferring the physical properties of yeast chromatin through Bayesian analysis of whole nucleus simulations. Genome Biol. 2017;18(1):81. 28468672 10.1186/s13059-017-1199-x PMC5414205

[40] Guilbaud S, Salome L, Destainville N, et al. Dependence of DNA persistence length on ionic strength and ion type. Phys Rev Lett. 2019;122(2):028102. 30720315 10.1103/PhysRevLett.122.028102

[41] Bonato A, Brackley CA, Johnson J, et al. Chromosome compaction and chromatin stiffness enhance diffusive loop extrusion by slip-link proteins. Soft Matter. 2020;16(9):2406–2414. 32067018 10.1039/c9sm01875a

[42] Yu M, Ren B. The three-dimensional organization of mammalian genomes. Annu Rev Cell Dev Biol. 2017;33(1):265–289. 28783961 10.1146/annurev-cellbio-100616-060531 PMC5837811

[43] Weeks JD, Chandler D, Andersen HC. Role of repulsive forces in determining the equilibrium structure of simple liquids. J Chem Phys. 1971;54(12):5237–5247.

[44] Marenduzzo D, Finan K, Cook PR. The depletion attraction: an underappreciated force driving cellular organization. J Cell Biol. 2006;175(5):681–686. 17145959 10.1083/jcb.200609066 PMC2064666

[45] Götzelmann B, Evens R, Dietrich S. Depletion forces in fluids. Phys Rev. 1998;57:6785.

[46] Arkın H, Janke W. Ground-state properties of a polymer chain in an attractive sphere. J Phys Chem B. 2012;116(34):10379–10386. 22823401 10.1021/jp304844k

[47] Newman M, Barkema G. Monte Carlo methods in statistical physics. Chapter 1-4. Oxford University Press, Oxford, UK; 1999.

[48] Metropolis N, Rosenbluth AW, Rosenbluth MN, et al. Equation of state calculations by fast computing machines. J Chem Phys. 1953;21(6):1087–1092. 10.1063/5.0309018 41342507

[49] Shaban HA, Barth R, Recoules L, et al. Hi-D: nanoscale mapping of nuclear dynamics in single living cells. Genome Biol. 2020;21(1):95. 32312289 10.1186/s13059-020-02002-6 PMC7168861

[50] Brachner A, Foisner R. Evolvement of LEM proteins as chromatin tethers at the nuclear periphery. Biochem Soc Trans. 2011;39(6):1735–1741. 22103517 10.1042/BST20110724 PMC4333761

[51] Towbin BD, Gonzalez-Aguilera C, Sack R, et al. Step-wise methylation of histone H3K9 positions heterochromatin at the nuclear periphery. Cell. 2012;150(5):934–947. 22939621 10.1016/j.cell.2012.06.051

[52] De Gennes PG. Scaling concepts in polymer physics. Ithaca, New York: Cornell University Press; 1979.

[53] Scheutjens JM, Fleer GJ. Statistical theory of the adsorption of interacting chain molecules. 2. Train, loop, and tail size distribution. J Phys Chem. 1980;84(2):178–190.

[54] Segura-Totten M, Kowalski AK, Craigie R, et al. Barrier-to-autointegration factor: major roles in chromatin decondensation and nuclear assembly. J Cell Biol. 2002;158(3):475–485. 12163470 10.1083/jcb.200202019 PMC2173821

[55] Malik P, Korfali N, Srsen V, et al. Cell-specific and lamin-dependent targeting of novel transmembrane proteins in the nuclear envelope. Cell Mol Life Sci. 2010;67(8):1353–1369. 20091084 10.1007/s00018-010-0257-2 PMC2839517

[56] Thanisch K, Song C, Engelkamp D, *et al*. Nuclear envelope localization of LEMD2 is developmentally dynamic and lamin A/C dependent yet insufficient for heterochromatin tethering. Differentiation. 2017;94:58–70. 28056360 10.1016/j.diff.2016.12.002

[57] Gonzalez-Sandoval A, Towbin BD, Kalck V, *et al*. Perinuclear Anchoring of H3K9-methylated chromatin stabilizes induced cell fate in C. elegans embryos. Cell. 2015;163(6):1333–1347. 26607792 10.1016/j.cell.2015.10.066

[58] Brunet A, Forsberg F, Fan Q, et al. Nuclear lamin B1 interactions with chromatin during the circadian cycle are uncoupled from periodic gene expression. Front Genet. 2019;10:917. 31632442 10.3389/fgene.2019.00917 PMC6785633

[59] Reddy KL, Zullo JM, Bertolino E, et al. Transcriptional repression mediated by repositioning of genes to the nuclear lamina. Nature. 2008;452(7184):243–247. 18272965 10.1038/nature06727

