# Supplementary material for: Physical constraints in polymer modeling of chromatin associations with the nuclear periphery at kilobase scale
Source: Nucleus. 2021 Jan 12;12(1):6–20. doi: 10.1080/19491034.2020.1868105 (PMC7808377; doi:10.1080/19491034.2020.1868105)
Supplement: Supplemental Material [file KNCL_A_1868105_SM3277.pdf]

Supplementary data

Supplementary information 1. Note on polymer persistence length.

Thermal fluctuations of a polymer depend on its persistence length L_P which defines its bending properties. For nucleic acids, L_P depends on DNA structure and the degree of chromatin compaction. Estimations of L_P for double-stranded DNA converge on ~50 nm, but for heterogeneous chromatin conformations L_P can cover a wide range, extending from ~50 to > 300 nm (refs. (1-20) below).

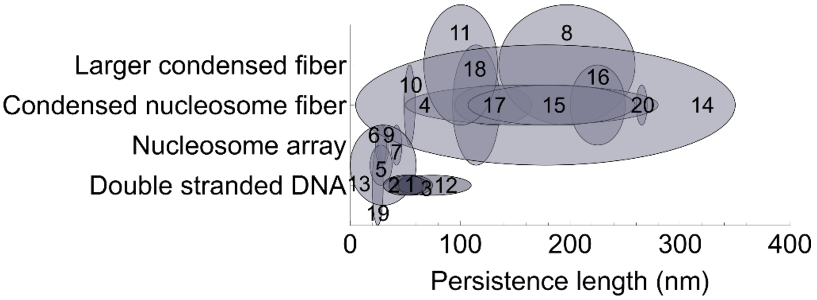

The diagram shows a qualitative representation of an overview of L_P values from the literature based on wet-lab data, *in silico* data or computational modeling, as a function of the degree of compaction of the chromatin fiber (diameter of the genomic object under study). Numbers indicate the reference number in the list below. Note that loosely packed chromatin, as double-stranded DNA or nucleosome arrays, display lower persistence lengths than more compact configurations. This implies that euchromatin typically harbors a lower persistence length, and is therefore more flexible, than heterochromatin.

<u>Supplementary References</u>

1. Manning, G. (2001) Counterion Condensation on a Helical Charge Lattice. *Macromolecules*, **34**, 4650-4655.
2. Brunet, A., Tardin, C., Salome, L., Rousseau, P., Destainville, N. and Manghi, M. (2015) Dependence of DNA persistence length on ionic strength of solutions with monovalent and divalent salts: a joint theory-experiment study. *Macromolecules*, **48**, 3641-3652.
3. Brunet, A., Salome, L., Rousseau, P., Destainville, N., Manghi, M. and Tardin, C. (2018) How does temperature impact the conformation of single DNA molecules below melting temperature? *Nucleic Acids Res*, **46**, 2074-2081.
4. Aumann, F., Lankas, F., Caudron, M. and Langowski, J. (2006) Monte Carlo simulation of chromatin stretching. *Phys Rev E Stat Nonlin Soft Matter Phys*, **73**, 041927.
5. Bancaud, A., Conde e Silva, N., Barbi, M., Wagner, G., Allemand, J.F., Mozziconacci, J., Lavelle, C., Croquette, V., Victor, J.M., Prunell, A. *et al.* (2006) Structural plasticity of single chromatin fibers revealed by torsional manipulation. *Nat Struct Mol Biol*, **13**, 444-450.
6. Bennink, M.L., Leuba, S.H., Leno, G.H., Zlatanova, J., de Grooth, B.G. and Greve, J. (2001) Unfolding individual nucleosomes by stretching single chromatin fibers with optical tweezers. *Nat Struct Biol*, **8**, 606-610.
7. Brower-Toland, B.D., Smith, C.L., Yeh, R.C., Lis, J.T., Peterson, C.L. and Wang, M.D. (2002) Mechanical disruption of individual nucleosomes reveals a reversible multistage release of DNA. *Proc Natl Acad Sci U S A*, **99**, 1960-1965.
8. Bystricky, K., Heun, P., Gehlen, L., Langowski, J. and Gasser, S.M. (2004) Long-range compaction and flexibility of interphase chromatin in budding yeast analyzed by high-resolution imaging techniques. *Proc Natl Acad Sci U S A*, **101**, 16495-16500.
9. Cui, Y. and Bustamante, C. (2000) Pulling a single chromatin fiber reveals the forces that maintain its higher-order structure. *Proc Natl Acad Sci U S A*, **97**, 127-132.
10. Dekker, J. (2008) Mapping in vivo chromatin interactions in yeast suggests an extended chromatin fiber with regional variation in compaction. *J Biol Chem*, **283**, 34532-34540.

11. Dekker, J., Rippe, K., Dekker, M. and Kleckner, N. (2002) Capturing chromosome conformation. *Science*, **295**, 1306-1311.
12. Guilbaud, S., Salome, L., Destainville, N., Manghi, M. and Tardin, C. (2019) Dependence of DNA Persistence Length on Ionic Strength and Ion Type. *Phys Rev Lett*, **122**, 028102.
13. Hajjoul, H., Mathon, J., Ranchon, H., Goiffon, I., Mozziconacci, J., Albert, B., Carrivain, P., Victor, J.M., Gadal, O., Bystricky, K. *et al.* (2013) High-throughput chromatin motion tracking in living yeast reveals the flexibility of the fiber throughout the genome. *Genome Res*, **23**, 1829-1838.
14. Kepper, N., Foethke, D., Stehr, R., Wedemann, G. and Rippe, K. (2008) Nucleosome geometry and internucleosomal interactions control the chromatin fiber conformation. *Biophys J*, **95**, 3692-3705.
15. Langowski, J. and Heermann, D.W. (2007) Computational modeling of the chromatin fiber. *Semin Cell Dev Biol*, **18**, 659-667.
16. Mergell, B., Everaers, R. and Schiessel, H. (2004) Nucleosome interactions in chromatin: fiber stiffening and hairpin formation. *Phys Rev E Stat Nonlin Soft Matter Phys*, **70**, 011915.
17. Munkel, C., Eils, R., Dietzel, S., Zink, D., Mehring, C., Wedemann, G., Cremer, T. and Langowski, J. (1999) Compartmentalization of interphase chromosomes observed in simulation and experiment. *J Mol Biol*, **285**, 1053-1065.
18. Ostashevsky, J.Y. and Lange, C.S. (1994) The 30 nm chromatin fiber as a flexible polymer. *J Biomol Struct Dyn*, **11**, 813-820.
19. Ringrose, L., Chabanis, S., Angrand, P.O., Woodroofe, C. and Stewart, A.F. (1999) Quantitative comparison of DNA looping in vitro and in vivo: chromatin increases effective DNA flexibility at short distances. *EMBO J*, **18**, 6630-6641.
20. Wedemann, G. and Langowski, J. (2002) Computer simulation of the 30-nanometer chromatin fiber. *Biophys J*, **82**, 2847-2859.

Supplementary information 2. Theoretical rigid approximation of bead-to-periphery distance.

We approximate the nuclear periphery (NP) as a locally flat surface limiting the motion of the chromatin polymer to, as shown here, the upper half space. Based on a worm-like chain polymer model, in a rigid regime, the chromatin chain behaves as a rigid rod. Thus the bead-to-NP distance (z) depends on the contour length of the chain (*L*) and the imposed offset above the surface (h = r_{bead} = 15 nm) for the first bead.

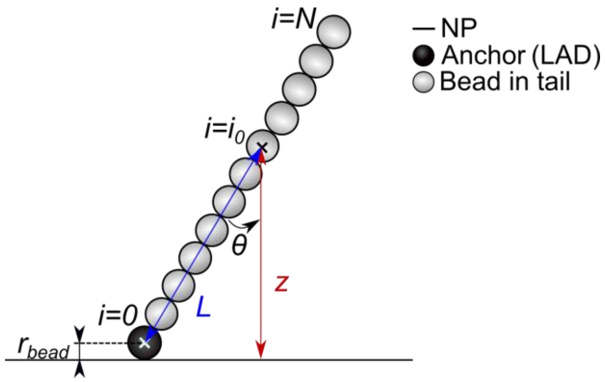

Representation of a rigid chain anchored by one end to NP.

L, the contour length of the subchain ranging from $i = 0$ to $i = i_0$, can be expressed as $L = 2i_0\, r_{bead}$.

In addition, bead-to-NP distance of the bead $i = i_0$, is equal to $z = L \cos\theta + r_{bead}$.

In the following, we note $D_{toNP}(i_0)$, the ensemble average bead-NP distance z. We integrated the expression of $D_{toNP}(i)$ on half the solid angles, expressing the accessible space by the chain in this specific geometry:

$$D_{toNP}(i_0) = \frac{1}{2\pi}\int sin\,\theta\; d\theta \int d\,\phi\; Lcos\theta + r_{bead}$$

Using sin 2θ = 2 sinθ cosθ leads to the following expression:

$$D_{toNP}(i_0) = r_{bead} + L\frac{1}{2}\int sin\,2\theta\; d\theta$$

$$D_{toNP}(i_0) = r_{bead} + \frac{L}{2}$$

Using $L=2i_0\, r_{bead}$ allows expression of $D_{toNP}(i_0)$ as a function of i_0 :

$$D_{toNP}(i) = (i_0 + 1)r_{bead}$$

Starting with bead indexing $i = 1$ leads to expression of bead-NP distances in a rigid approximation regime:

$$D_{toNP}(i) = ir_{bead}$$

Supplementary Figures

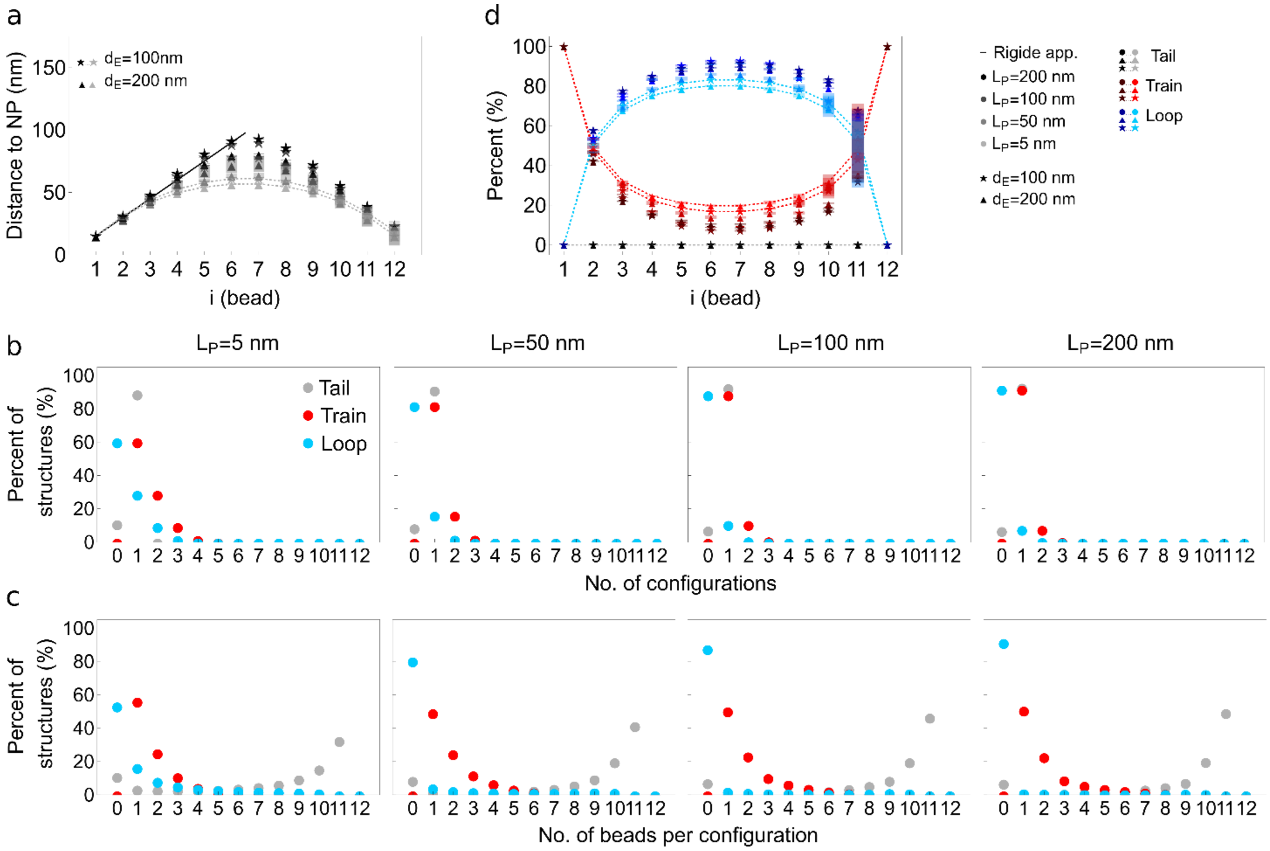

Figure S1. Behavior of a chromatin chain at a neutral surface NP. (a) Mean distance from bead center to NP along a chain pinned with 2 anchors (beads 1 and 12) to NP, with (**i**) d_E = 100 and 200 nm between them. Black line represents the theoretical rigid approximation (see Supplemental information 1). (b,c) Polymer configurations with one anchor. Percentage of structures (y axis) with (b) a given number of tail-train-loop configurations (x axis) and (c) with a given number of beads in each configuration (x axis). Data are shown for increasing polymer stiffness L_P. (d) Proportion of structures with a tail, train, loop configuration for a polymer with 2 anchor points (bead 1, 12) as a function of bead position along the chain (x axis) and L_P (color intensity; legend). In (a) and (d), lines connect data points for L_P = 5 nm for clearer visualization of the trends. See also legend to **Figure 2** (in reference to panel a) and **Figure 3** (in reference to panels b-d) for biological implications of the findings.

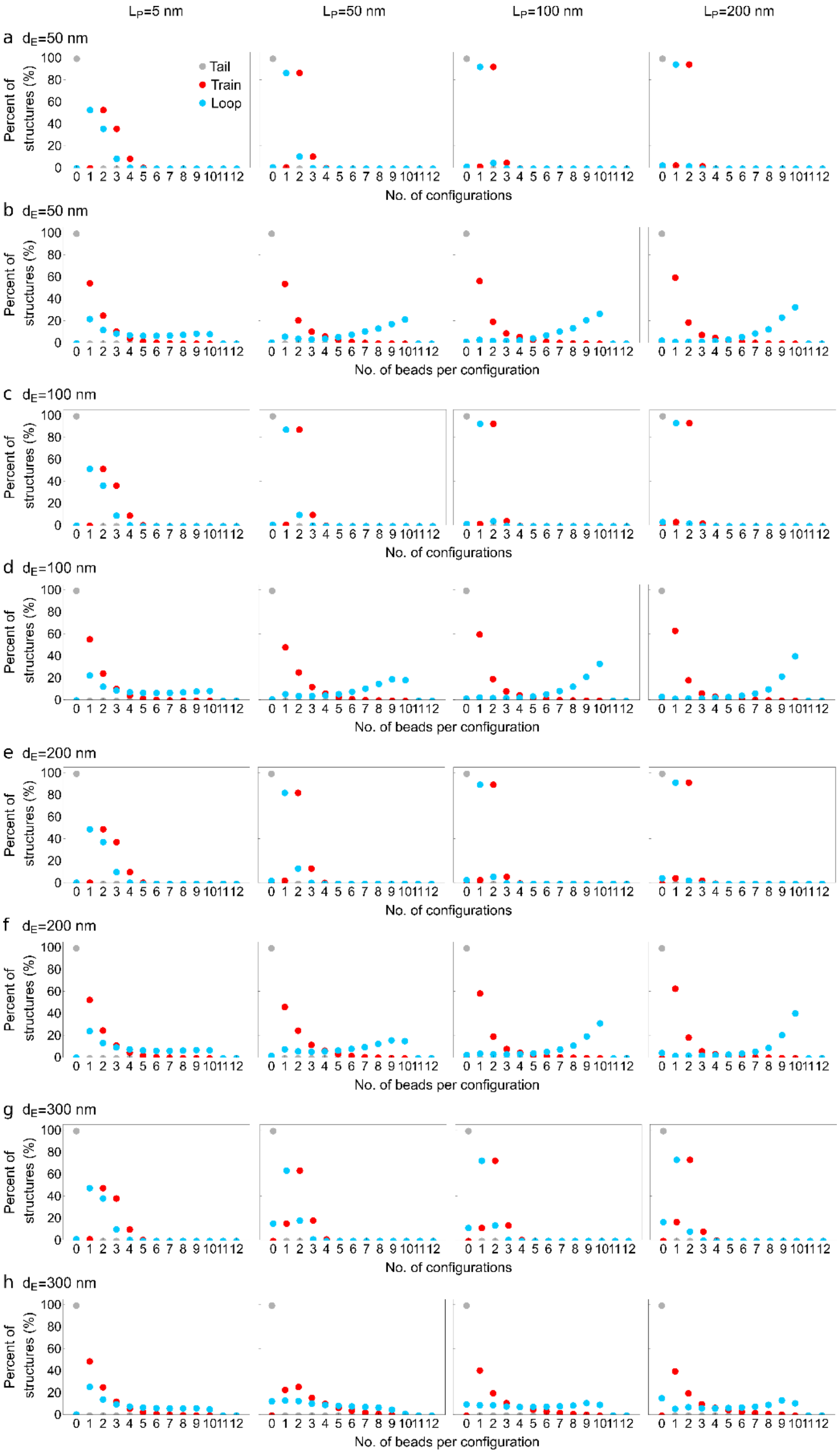

Figure S2. Polymer configurations with two anchor points at NP. Percentage of structures (y axis) with (a,c,e,g) a given number of tail-train-loop configurations (x axis) and (b,d,f,h) a given number of beads in each configuration (x axis). Data are shown for increasing L_P and d_E between anchors. See also legend to **Figure 3e,f** for more details and legend to **Figure 3** as a whole for biological implications.

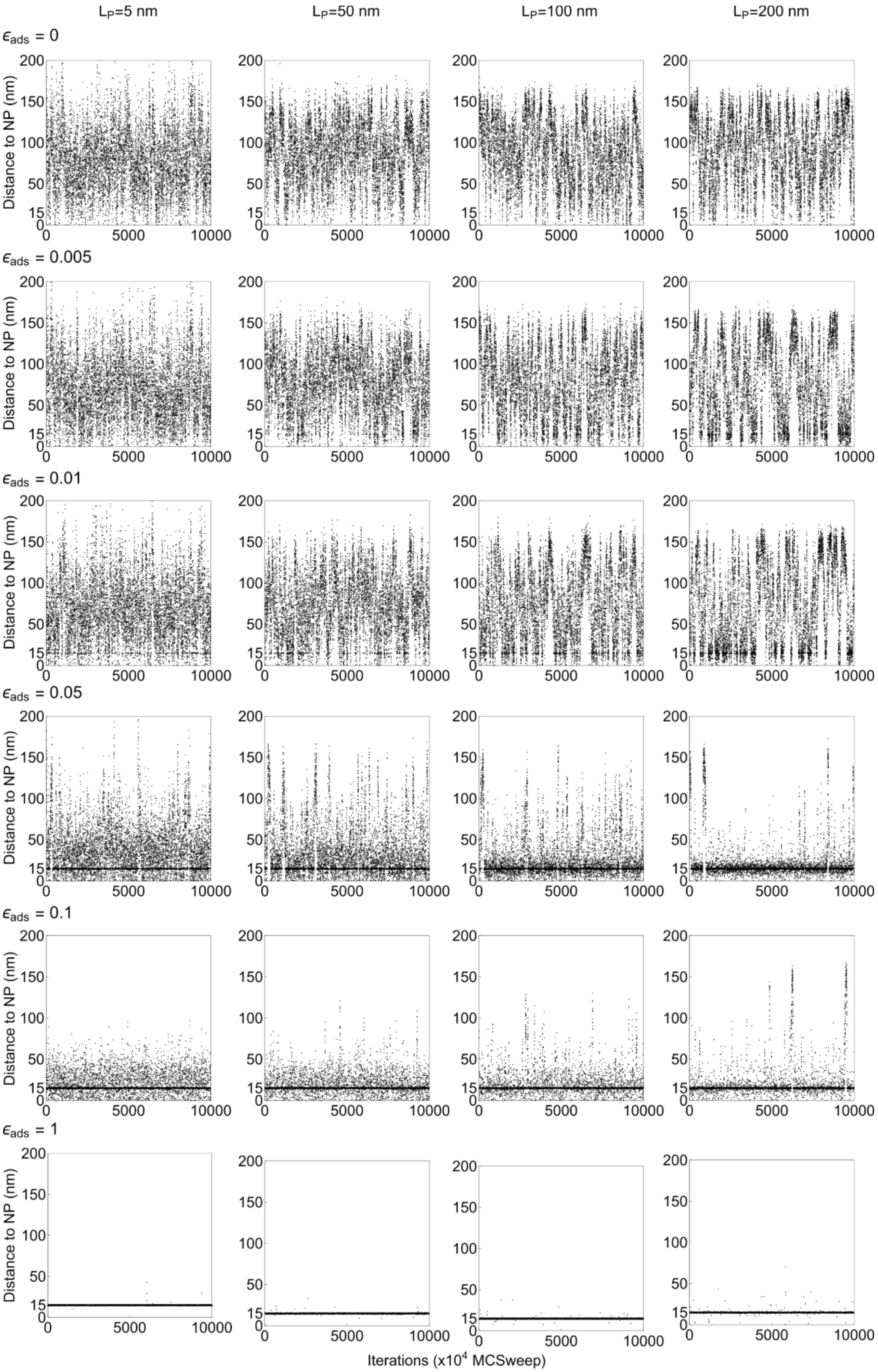

Figure S3. Examples of bead trajectories as distances to NP (y axis) across simulations in a polymer with one anchor to the surface NP, as a function of surface attraction potential ε_{ads} and polymer stiffness L_P. Data are shown for bead *i* = 6 (in the middle of the chain). Minimal distance to NP is 15 nm (one bead radius; y axis), which is pre-set in our models (see Supplemental information 2).

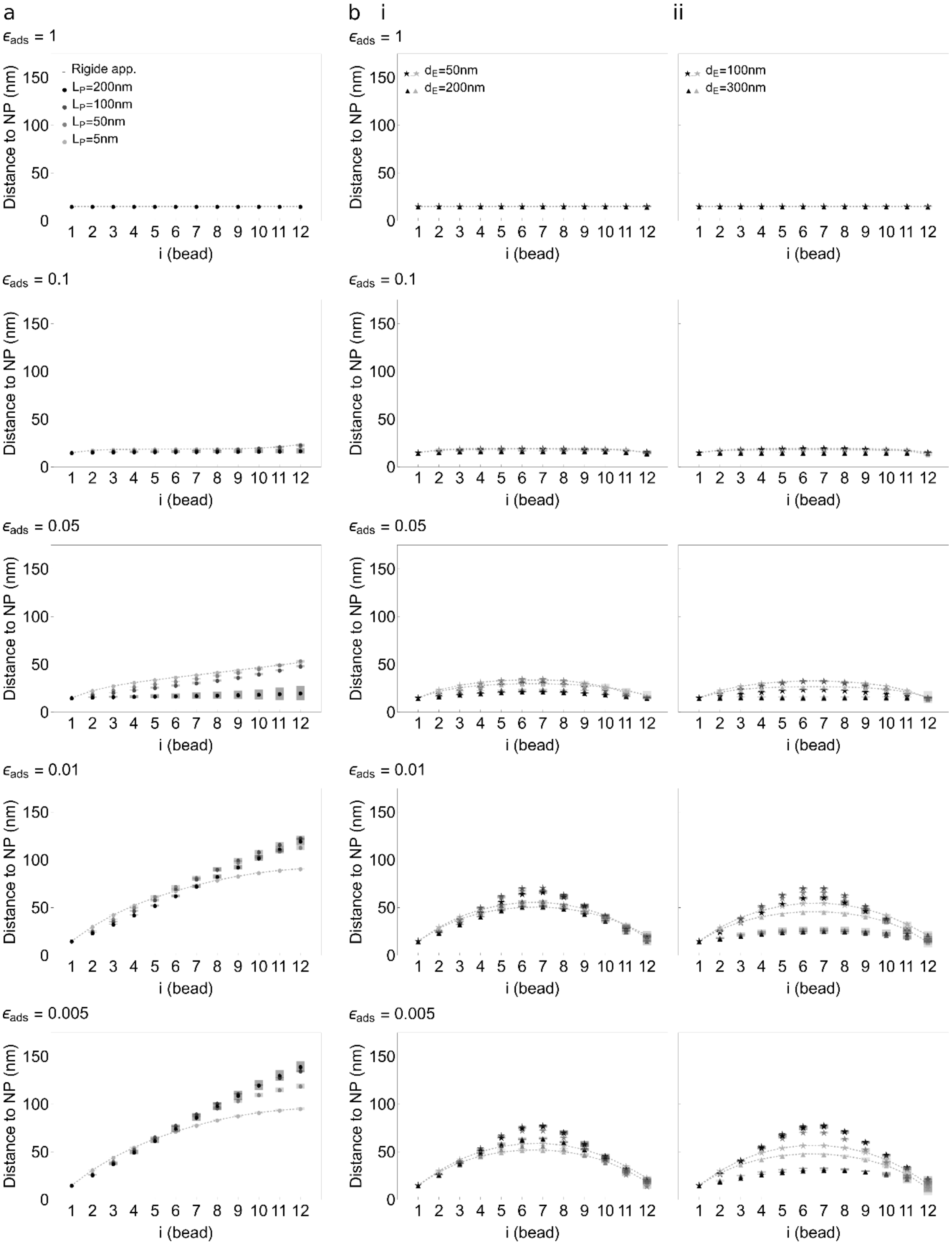

Figure S4. Behavior of a chromatin polymer at a surface NP fitted with a varying attraction potential towards the polymer. (a,b) Mean distances from bead center to NP for polymers with (a) one anchor and (b) two anchors to NP. Data are shown as a function of chain stiffness L_P (gray scale) and Euclidian distance d_E between the two anchor points (in bi and bii), for decreasing attraction potentials ε_{ads}. Gray scale legend in (a) is for all panels in (a) and (b). In (a) and (b), lines connect data points for L_P = 5 nm for clearer visualization of the trends.

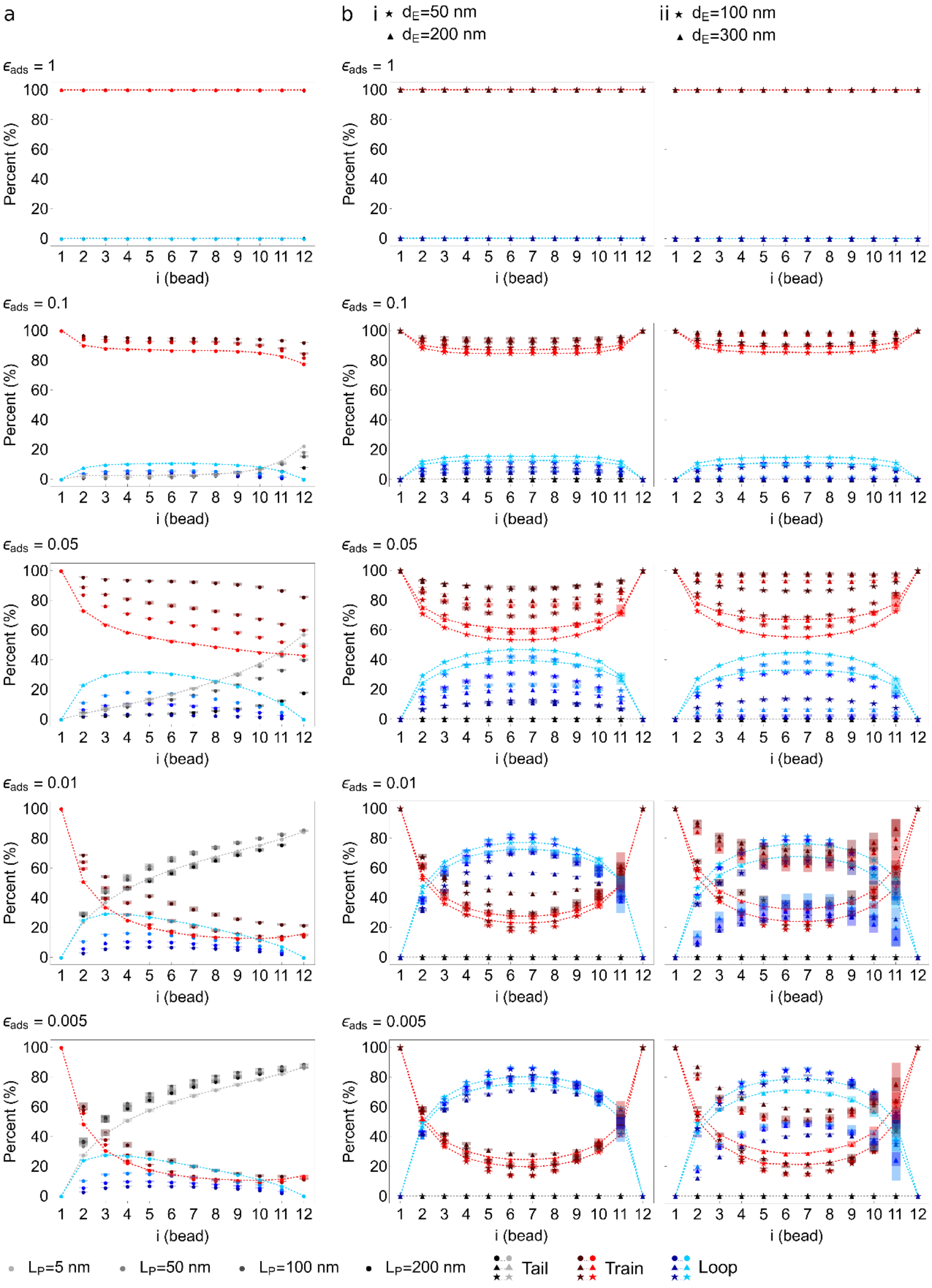

Figure S5. Tail-train-loop configurations of a polymer at a surface NP fitted with a varying attraction potential. (a) Percentage of beads in tail-train-loop configurations in simulations with one anchor as a function of bead position along the chain (x axis), attraction potential (0.005-1) and L_P (legend). (b) Same as in (a) with two anchors (bead 1, 12) and as a function of d_E between anchors: (i) d_E = 50 and 200 nm; (ii) d_E = 100 and 300 nm. In (a) and (b), lines connect data points for L_P = 5 nm for clearer visualization of the trends. See also **Figure 5** for biological implications.

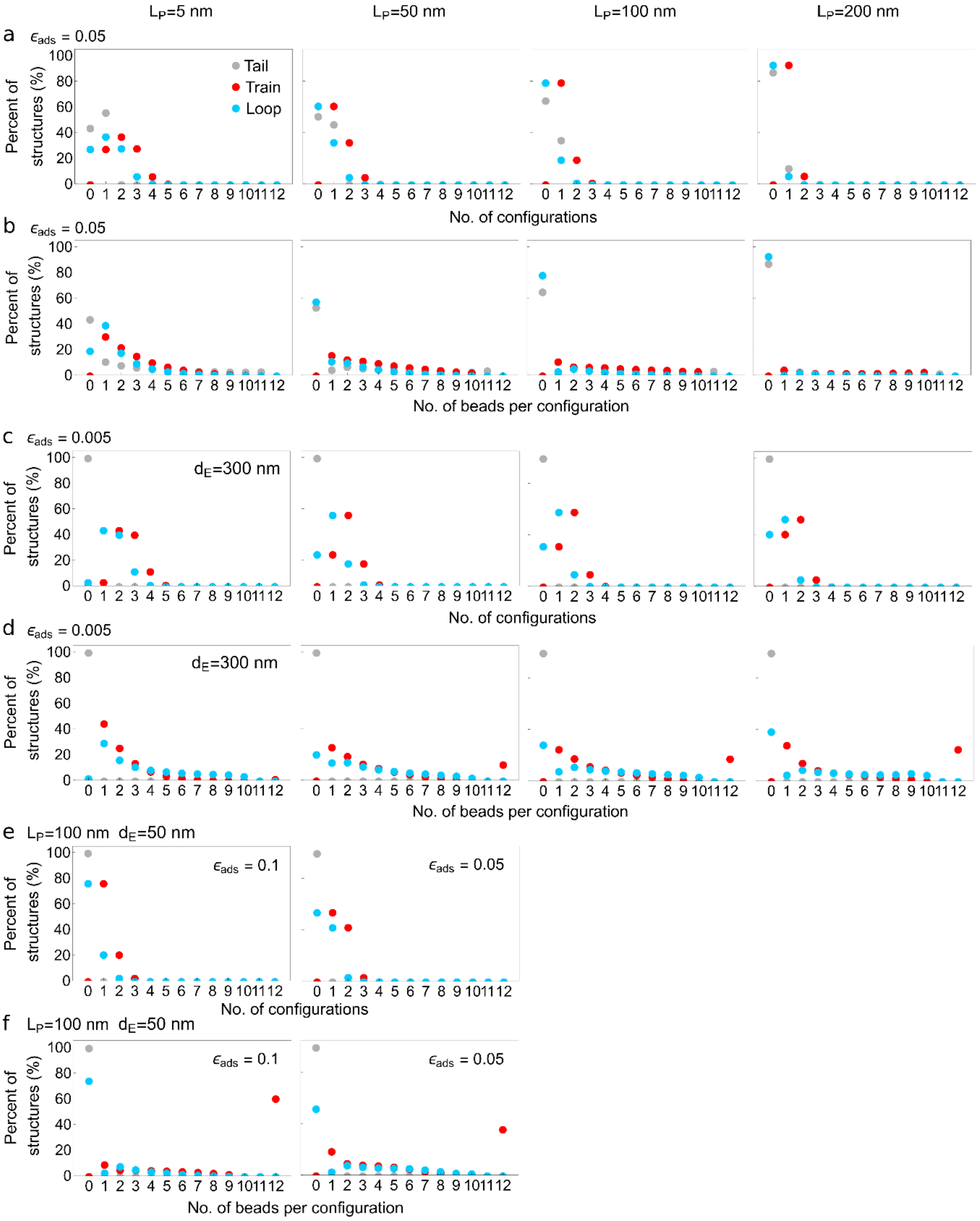

Figure S6. Polymer configurations with (a,b) one anchor and (c,d) two anchors at a surface NP fitted with a varying attraction potential. Percentage of one-anchor structures with a given tail-train-loop configuration, with (a,c) a given number of configurations (x axis) and (b,d) a given number of beads in these configurations (x axis), for increasing polymer stiffness L_P. Data are shown for attraction potential (a,b) ε_{ads} = 0.05 and (c,d) ε_{ads} = 0.005 with a Euclidian distance d_E = 300 nm between the two anchors. (e,f) Same as (c,d) with L_P = 100 nm, d_E = 50 nm and ε_{ads} = 0.1 or 0.05; see Discussion in the main text.
